# Antioxidant Therapy Reduces Oxidative Stress, Restores Na,K-ATPase Function and Induces Neuroprotection in Rodent Models of Seizure and Epilepsy: A Systematic Review and Meta-Analysis

**DOI:** 10.3390/antiox12071397

**Published:** 2023-07-07

**Authors:** Anderson Dutra de Melo, Victor Antonio Ferreira Freire, Ítalo Leonardo Diogo, Hérica de Lima Santos, Leandro Augusto Barbosa, Luciana Estefani Drumond de Carvalho

**Affiliations:** 1 Departamento de Ciências e Linguagens, Instituto Federal de Minas Gerais, Bambui 38900-000, Minas Gerais, Brazil; anderson.melo@ifmg.edu.br; 2 Laboratório de Bioquímica Celular, Universidade Federal de São João Del Rei, Divinopolis 35501-296, Minas Gerais, Brazil

**Keywords:** Na,K-ATPase, oxidative stress, epilepsy, exogenous antioxidants

## Abstract

Epilepsy is a neurological disorder characterized by epileptic seizures resulting from neuronal hyperexcitability, which may be related to failures in Na,K-ATPase activity and oxidative stress participation. We conducted this study to investigate the impact of antioxidant therapy on oxidative stress, Na,K-ATPase activity, seizure factors, and mortality in rodent seizure/epilepsy models induced by pentylenetetrazol (PTZ), pilocarpine (PILO), and kainic acid (KA). After screening 561 records in the MEDLINE, EMBASE, Web of Science, Science Direct, and Scopus databases, 22 were included in the systematic review following the PRISMA guidelines. The meta-analysis included 14 studies and showed that in epileptic animals there was an increase in the oxidizing agents nitric oxide (NO) and malondialdehyde (MDA), with a reduction in endogenous antioxidants reduced glutathione (GSH) and superoxide dismutase (SO). The Na,K-ATPase activity was reduced in all areas evaluated. Antioxidant therapy reversed all of these parameters altered by seizure or epilepsy induction. In addition, there was a percentage decrease in the number of seizures and mortality, and a meta-analysis showed a longer seizure latency in animals using antioxidant therapy. Thus, this study suggests that the use of antioxidants promotes neuroprotective effects and mitigates the effects of epilepsy. The protocol was registered in the Prospective Register of Systematic Reviews (PROSPERO) CRD42022356960.

## 1. Introduction

Epilepsy is a chronic neurological disorder that affects more than 70 million people worldwide, with a generally bimodal incidence that mainly affects children and the elderly [1]. It is characterized by a persistent propensity to develop epileptic seizures due to abnormal excessive or synchronous neuronal activity in the brain [2]. The molecular mechanisms underlying the development of epilepsy are related to neural injury resulting from several factors, including oxidative stress (OS) in neural tissues [3,4,5,6].

Oxidative stress occurs when the antioxidant defense system, consisting of endogenous antioxidants, such as catalase (CAT), glutathione peroxidase (GPx), glutathione reductase (GR), superoxide dismutase (SOD), and reduced glutathione (GSH), and a variety of exogenous antioxidants obtained from the diet, cannot eliminate excess reactive species (RS) which are the result of cellular metabolism and/or external factors [7,8,9,10,11].

The combination of excessive reactive oxygen species (ROS) production with nitric oxide (NO) to form peroxynitrite, a reactive nitrogen species, leads to lipid peroxidation and direct protein damage, which alters membrane and cell functions [12,13,14]. This fact makes the brain highly susceptible to oxidative stress because of its high metabolism and high concentrations of unsaturated fatty acids [15].

As a consequence of oxidative metabolism and lipid peroxidation, damage occurs to the constituents of neuronal membranes, including Na,K-ATPase (NKA) [16,17]. This enzyme maintains the electrical activity of excitable cells and is essential for the maintenance of cellular homeostasis, as it is responsible for the ionic composition within the cells and osmotic balance [18,19]. Consequently, failures in the NKA activity can interfere with cell excitability and facilitate the onset or propagation of a seizure [20,21].

In order to improve antioxidant defense, antioxidant therapy can be performed through the ingestion of substances with antioxidant activity, either through an adequate diet or through supplementation [22,23,24]. Several studies report benefits with the use of antioxidant therapy in animal models of neurological disorders [25,26,27,28] and in human studies [29,30,31]. Preclinical studies have indicated that antioxidant therapy reduces oxidative damage and epileptogenic potential [32,33,34]. However, clinical studies are controversial. Studies have shown a reduction in seizures [35] and oxidative stress [36,37]. Other studies have indicated no significant changes in seizure frequency [38].

Animal models have been widely used for the study of epilepsy, and the most commonly used proconvulsants to induce this model are pilocarpine (PILO), kainic acid (KA), and pentylenetetrazole (PTZ). PILO and KA induce a model of chronic temporal lobe epilepsy, which affects some neural substrates such as the hippocampus and cortex [39,40,41,42,43]. Pilocarpine is a cholinergic agonist and kainic acid is an l-glutamate analogue and an agonist of AMPA and kainic acid receptors; both induce seizures that progress to epilepsy [40,41,42]. PTZ, used as an acute seizure model, induces generalized seizures by decreasing GABAergic function through antagonism of GABAa receptors [44]. All these models allow the study of postictal abnormalities.

Considering the importance of epilepsy and the therapeutic potential of antioxidants, we conducted a systematic review and meta-analysis of preclinical studies that investigated the action of different antioxidants in animal models of seizures or epilepsy. The aim of this study was to investigate the effects of different antioxidant therapies on oxidative stress and Na,K-ATPase activity in neural tissue in a rodent model of seizures or epilepsy and to verify the impact of this use on seizure parameters and mortality. In this way, advances can be made in the knowledge related to biochemical factors, safety, and neuroprotection on the use of these antioxidants in preclinical studies of seizures and epilepsy, which will enable future clinical studies.

## 2. Materials and Methods

This review was conducted in accordance with the Preferred Reporting Items for Systematic Reviews and Meta-Analyses (PRISMA) [45]. The PICO and PECO were used because this study was divided into Intervention and Exposure groups. Thus, we have Population (P)—rats and mice; Intervention (I)—antioxidant therapy or Exposure (E)—epilepsy; Comparison (C)—control or sham animals; and Outcomes (O)—NKA activity, oxidative stress, and behavioral changes. The protocol was registered in the International Prospective Register of Systematic Reviews (PROSPERO) CRD42022356960.

### 2.1. Research Strategy and Selection of Studies

To verify the effect of antioxidant therapy on NKA activity in experimental models of epilepsy, a systematic search was performed using MEDLINE (PubMed), EMBASE, Web of Science, Science Direct, and Scopus, regardless of publication date.

The following descriptors that could be in the titles, abstracts, and/or keywords were used: for epilepsy (epileps*, “epilepsy seizure”, convulsion, seizure), for Na,K-ATPase (“sodium, potassium exchanging ATPase”, “sodium potassium ATPase”, “sodium potassium adenosinetriphosphatase”, “ATPase sodium potassium”, “Na^+^K^+^ transporting ATPase”, “Na^+^K^+^ exchanging ATPase”, “Na^+^K^+^-ATPase”, “sodium pump”, “sodium potassium pump”, “adenosine triphosphatase”, “Na/K-ATPase”), and for oxidative stress combined with antioxidants (“oxidative stresses”, “oxidative stress”, “nitro oxidative stress”, “nitro-oxidative stress”, “oxidative injury”, “oxidative damage”, antioxidants, anti-oxidant, “antioxidant activity”). Among the descriptions the term OR was used and among the three groups of descriptors combined with each other, the term AND was used. The beginning and end of the study searches were 1 and 3 March 2022, respectively, with updates between 5 and 7 December 2022.

The eligibility criteria for the inclusion of studies were as follows: (1) The experimental models of epilepsy must be rats or mice. (2) Epilepsy can be chemically induced only by PILO, PTZ, or KA. (3) The study must analyze the activity of NKA in brain tissues and part of the epileptic animals should receive one antioxidant regardless of dosage and route of administration. (4) Experimental studies should include control animals or sham animals for comparison purposes. (5) Articles should be published in English, Portuguese, or Spanish. The following were excluded: (1) studies that used rats or mice with other associated pathologies and (2) literature review articles, abstracts, conference presentations, and book chapters.

After identifying studies in the databases, the articles were imported into the Rayyan software [46]. Duplicate studies were removed, and screening was performed by two blinded reviewers who independently evaluated the titles and abstracts of the studies. If there was disagreement, a third reviewer participated. After this procedure, the eligibility criteria were applied to the full articles, judging by inclusion or exclusion from this systematic review and meta-analysis. All studies that met the eligibility criteria but did not provide sufficient information, such as the sample size, were excluded from the meta-analysis; however, they were included in the systematic review.

### 2.2. Data Extraction and Assessment of Risk of Bias

One author (AM) extracted all data, which were verified by a second author (ID). Any disagreements were resolved by a third author (LC). Data extracted from primary studies were summarized in this systematic review and evaluated in the meta-analysis when at least 3 analyses were available: NKA activity, enzymatic antioxidant activity (superoxide dismutase—SOD, catalase—CAT, glutathione peroxidase—GPx, glutathione reductase—GR, glutathione S-transferase—GST), and GSH and oxidizing agents (malondialdehyde—MDA, protein carbonyl—PCA, nitric oxide—NO, RS). These data were considered when extracted from brain regions, and biochemical data extracted from the blood were not considered. Seizure latency, the number of seizures, and mortality were also recorded. WebPlotDigitizer software, version 4.5 [47], was used to collect the data available only in the graphs.

In addition, the following information was collected: (1) name of the first author and year of publication; (2) animal model, sex, and weight; (3) epilepsy-inducing drug, as well as the dosage and route of administration; (4) sample size of each experimental group; (5) type of antioxidant, as well as its dosage, form of administration, and duration of use; and (6) name of all outcomes.

Risk of bias assessment was performed using the Systematic Review Center for Laboratory Animal Experimentation (SYRCLE) tool by two independent reviewers. Possible sources of bias were verified in all studies included in this systematic review and divided into six large groups: selection, performance, detection, attrition, information, and other sources of bias. Details of the issues related to the inquiries are available in Supplementary Data N° 1.

### 2.3. Data Analysis

All data were analyzed using Revision Manager software [48]. The statistical method used was inverse variance, with random effects as the model of analysis, and the difference in the standardized mean as a measure of effect. For all analyses, statistical significance was set at *p* < 0.05, which was considered statistically significant [49]. The I^2^ statistic was used to assess heterogeneity, considering heterogeneity ≤40% as unimportant, 30–60% as moderate, and ≥75% as considerable [50]. Studies that presented data expressed as mean ± standard error (SEM) were converted into mean ± standard deviation (SD) using the following formula: SD = √n × standard error. 

## 3. Results

### 3.1. Search Results

**Figure 1:**
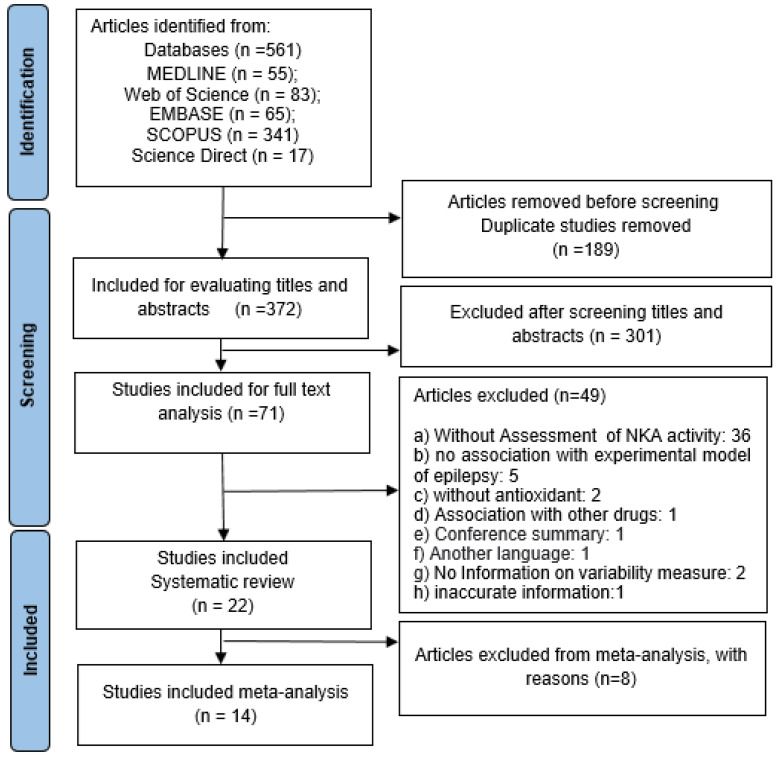
Flow diagram of study selection. Search process using the PRISMA flow diagram.

A total of 561 studies were identified from the five databases. After the removal of duplicate articles and screening of titles and abstracts, only the full text of 71 studies were analyzed. After this full-text analysis, 22 studies were included in the systematic review, of which 14 were included in the meta-analysis (Figure 1). The number of studies entered for each outcome and the location of the brain area analyzed are shown in Supplementary Data N° 2.

### 3.2. Assessment of the Risk of Bias

The results of the risk of bias assessment are presented in Table 1. The risks of selection bias, performance, detection, attrition, information, and others were evaluated and divided into ten questions. Of the 22 primary studies analyzed, 50% had a low risk of selection bias, whereas 16.7% had a high risk of bias. On the other hand, 52.3% of the studies were inaccurate regarding the risk of performance bias, while 47.7% were considered low risk. Detection and attrition biases each had a high risk of bias of 50%. Regarding information bias and other biases, there was a predominance of a low risk of bias (100% and 81.8%, respectively).

### 3.3. Animal and Antioxidant Characteristics of the Included Studies

The primary studies are summarized in Table 2 and were published between 2004 and 2022. Most studies (54.5%) used Wistar rats, followed by Swiss mice (36.4%), and Sprague Dawley rats (9.1%). All males had weights ranging from 150 to 300 g, except for one study that used animals of 21 days weighing between 40 and 50 g. In addition, the weights of the mice ranged from 18 g to 35 g. The sample size of each group ranged from five to fourteen animals for biochemical analysis.

Among these studies, only one used 10 mg/kg KA as an epilepsy-inducing drug. PILO was used in eight studies at dosages ranging from 300 to 400 mg/kg. PTZ was used in 13 articles, with dosages from 1.8 µmol to 90 mg/Kg. The main route of administration was the intraperitoneal route, which corresponded to 90.9%, in addition to the intrastriatal route in 9.1% of the studies.

Twenty different antioxidants were used in these studies, and only lipoic acid, creatine, and GM1 are considered endogenous antioxidants, while the others are classified as exogenous antioxidants. The dosages used in the primary studies varied between 10 and 300 mg/kg; 63.6% used the oral route to administer antioxidants, while the other studies used the intraperitoneal route. Furthermore, the majority of the animals were administered antioxidants between 30 and 60 min before the induction of epilepsy (Table 2).

### 3.4. Biochemical Parameters

Table 2 presents the summarized data of the biochemical parameters, showing the results obtained in each study included in the Systematic Review and separating the data obtained in the epilepsy and epilepsy + antioxidant groups.

#### 3.4.1. Oxidizing Agent

This systematic review collected data from studies evaluating oxidizing agents in the whole brain, hippocampus, cortex, and striatum. In the whole brains of epileptic animals, an increase in RS, NO, and MDA was observed in all analyses. With the use of exogenous antioxidants, there was a reduction in most of the RS (88.9%), NO (75%), and MDA (68.4%) levels. Studies in the hippocampus of epileptic animals showed that, in seven analyses of MDA, six showed an increase. This increase also occurred in PCA, RS, and NO analyses. The use of exogenous antioxidants indicates that in most analyses of PCA, NO, and MDA, there is a reduction in their levels in the hippocampus. This was also observed in the three analyses of RS. Furthermore, in the cortex of epileptic animals, there was an 80% increase in PCA analyses, in all MDA analyses, and in one study with RS. Regarding antioxidant treatment, there was a reduction in MDA and PCA levels by 100% and 66.7%, respectively. Regarding the RS data, in three analyses only one showed a reduction. In the striatum region, which showed an increase in MDA and PCA in epileptic animals, after treatment with exogenous antioxidants it only reduced in one PCA and in MDA analysis (Table 2).

**Figure 2:**
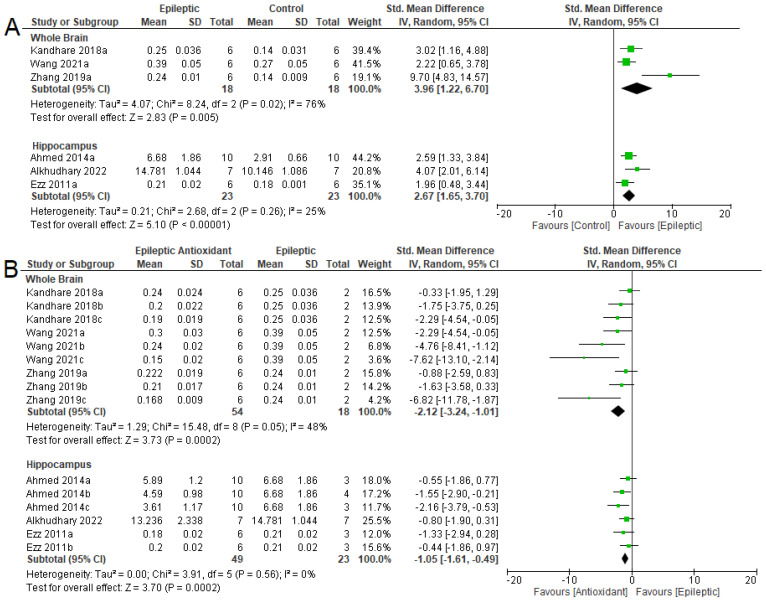
Forest plot comparing NO levels in different parts of the brain. (**A**) Effect of epilepsy versus control; (**B**) effect of intervention with exogenous antioxidants (epileptics/antioxidants versus epileptics); 95% confidence interval (CI); inverse variance (IV); standard deviation (SD); green square represents effect size. A negative standardized mean difference (SMD) represents lower levels of NO, whereas a positive SMD represents higher levels of NO [57,62,64,65,68,70].

The meta-analysis confirmed that there was an increase in NO levels in the whole brain (SMD = 3.96 [95% CI: 1.22 to 6.70] *p* = 0.005, I^2^ = 76%) and the hippocampus (SMD = 2.67 [95% CI: 1.65 to 3.70] *p* < 0.001, I^2^ = 25 %) of epileptic animals (Figure 2A). Furthermore, the use of antioxidants (Figure 2B), there was a reduction in NO levels in the whole brain (SMD = −2.12 [95% CI: −3.24 to −1.01], *p* = 0.0002, I^2^ = 48%) and hippocampus (SMD = −1.05 [95% CI: −1.61, −0.49], *p* = 0.0002, I^2^ = 0%).

**Figure 3:**
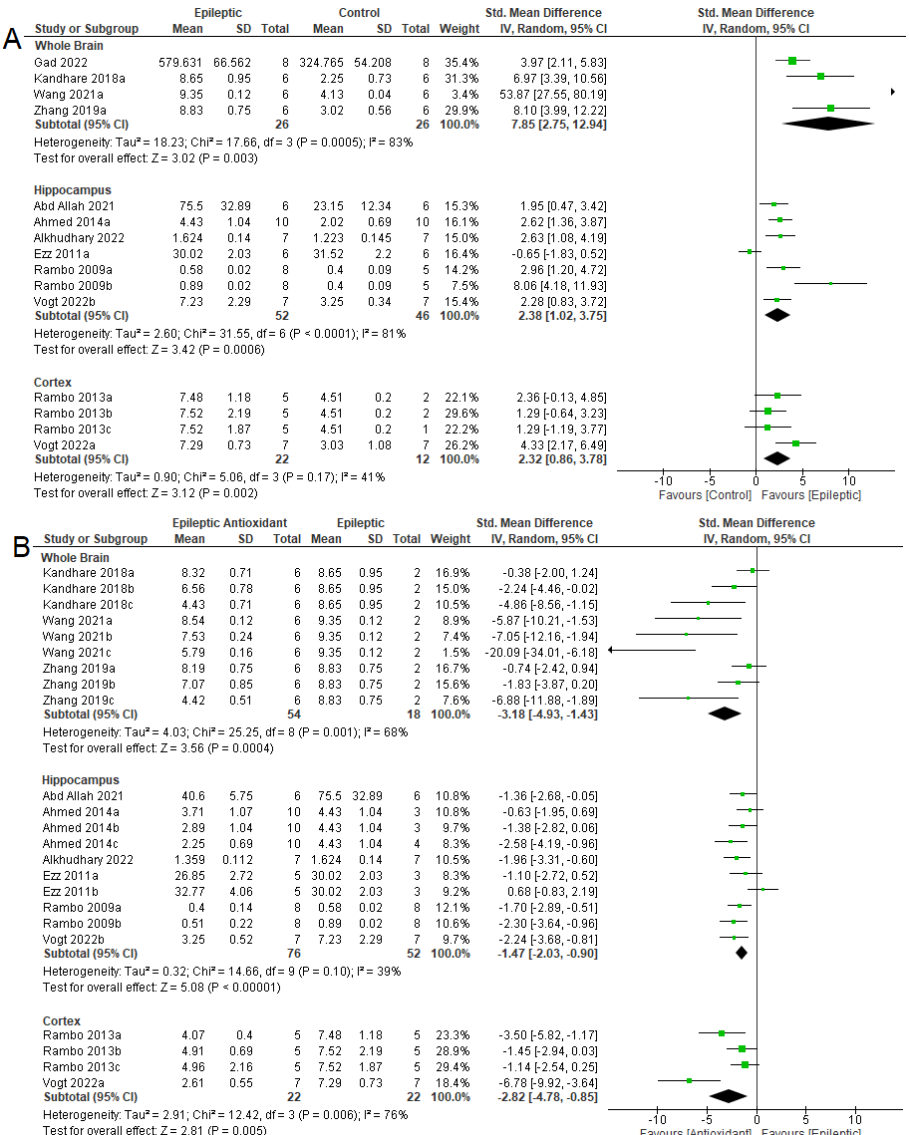
Forest plot comparing MDA levels in different parts of the brain. (**A**) Effect of epilepsy versus control; (**B**) effect of intervention with exogenous antioxidants (epileptics/antioxidants versus epileptics); 95% confidence interval (CI); inverse variance (IV); standard deviation (SD); green square represents effect size. A negative standardized mean difference (SMD) represents lower levels of MDA, whereas a positive SMD represents higher levels of MDA [54,57,61,62,64,65,67,68,69,70,71].

Regarding MDA levels, the meta-analysis indicated an increase in epilepsy in the three areas evaluated (Figure 3A). In the whole brain, the data were (SMD = 7.85 [95% CI: 2.75 to 12.94] *p* = 0.003, I^2^ = 83%). In the hippocampus and cortex, the results obtained were (SMD = 2.38 [95% CI: 1.02 to 3.75] *p* < 0.001, I^2^ = 81%) and (SMD = 2.32 [95% CI: 0.86 to 3.78] *p* = 0.002, I^2^ = 41%), respectively. With the use of antioxidants, there was a reduction in MDA levels in the three areas evaluated in epileptic animals treated with exogenous antioxidants (Figure 3B). For the whole brain, the following results were observed (SMD = −3.18 [95% CI: −4.93 to −1.43] *p* < 0.001, I^2^ = 68%). In the hippocampus and cortex, they were (SMD = −1.47 [95% CI: −2.03 to −0.90] *p* < 0.001, I^2^ = 39%) and (SMD = −2.82 [95% CI: −4.78 to −0.85] *p* = 0.005, I^2^ = 76%), respectively.

#### 3.4.2. Antioxidant System

The survey of primary studies conducted through the systematic review indicated that the antioxidant system was evaluated in five areas: the whole brain, hippocampus, cortex, striatum, and cerebellum (Table 2).

In the whole brain of untreated epileptic animals, there was a reduction in GSH levels and in all analyses of SOD, GR, and GPx activities, with an increase in GST and CAT activities in the two analyses. With antioxidant therapy, there was an increase in GSH levels and SOD and GR activity by 83.3%, 81.3%, and 100% of the analyses, respectively. However, there were no differences in the activities of GST (88.9%), CAT (70%), and GPx (100%). In the hippocampi of epileptic animals, there were reductions in GSH levels and GR activity in the analyses performed, whereas CAT activity was reduced in 66.6% of the analyses. Of the five SOD activity analyses, only two showed a reduction in the number of animals with epilepsy. GPx activity was assessed in three studies, with a reduction in only one of them. The use of antioxidants increased GSH levels and SOD and GPx activities by 57.1%, 60%, and 50%, respectively. It also increased in only one of two analyses. The use of antioxidants increased CAT activity in the three analyses but had no effect in the other three evaluations. In the cortex, only one study has evaluated SOD, GPx, and GSH levels in animals with epilepsy. There was an increase in SOD activity and a reduction in GSH levels, but no statistical difference in GPx activity. Regarding the use of exogenous antioxidants, it was observed that there was an increase in GSH levels with a reduction in SOD activity, whereas there was no effect on GPx activity (Table 2).

**Figure 4:**
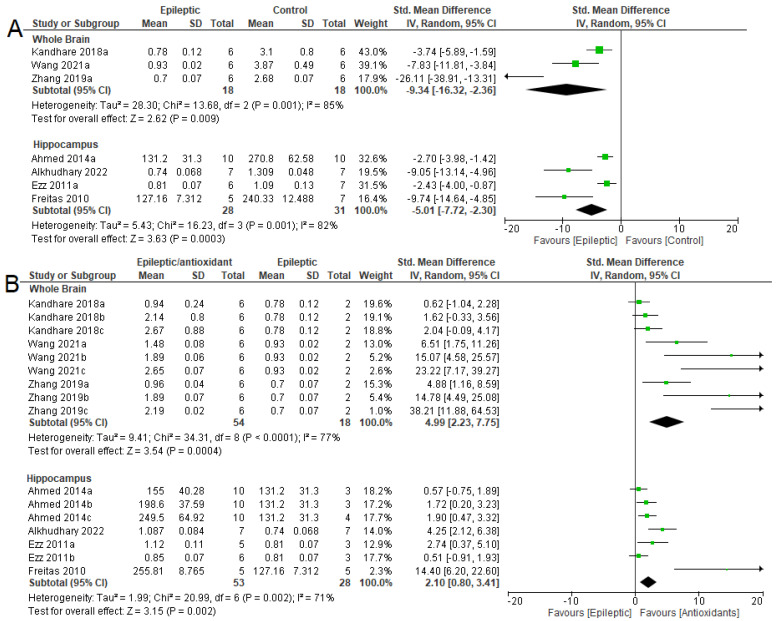
Forest plot comparing GSH levels in different parts of the brain. (**A**) Effect of epilepsy versus control; (**B**) effect of intervention with exogenous antioxidants (epileptics/antioxidants versus epileptics); 95% confidence interval (CI); inverse variance (IV); standard deviation (SD); green square represents effect size. A negative standardized mean difference (SMD) represents lower levels of GSH, whereas a positive SMD represents higher levels of GSH [20,57,62,64,65,68,70].

From the studies from which the meta-analysis could be performed, it was observed that in the whole brain (SMD = −9.34 [95% CI: −16.32 to −2.36], *p* = 0.009, I^2^ = 85%) and hippocampus (SMD = −5.01 [95% CI: −7.72 to −2.30] *p* = 0.0003, I^2^ = 82%) there was a reduction in GSH levels in epileptic animals (Figure 4A). There were increases in GSH levels in the brains of epileptic animals after antioxidant therapy (Figure 4B) (SMD = 4.99 [95% CI: 2.23 to 7.75] *p* = 0.0004, I^2^ = 77%) and the hippocampi (SMD = 2.10 [95% CI: 0.80 to 3.41] *p* = 0.002, I^2^ = 71%).

**Figure 5:**
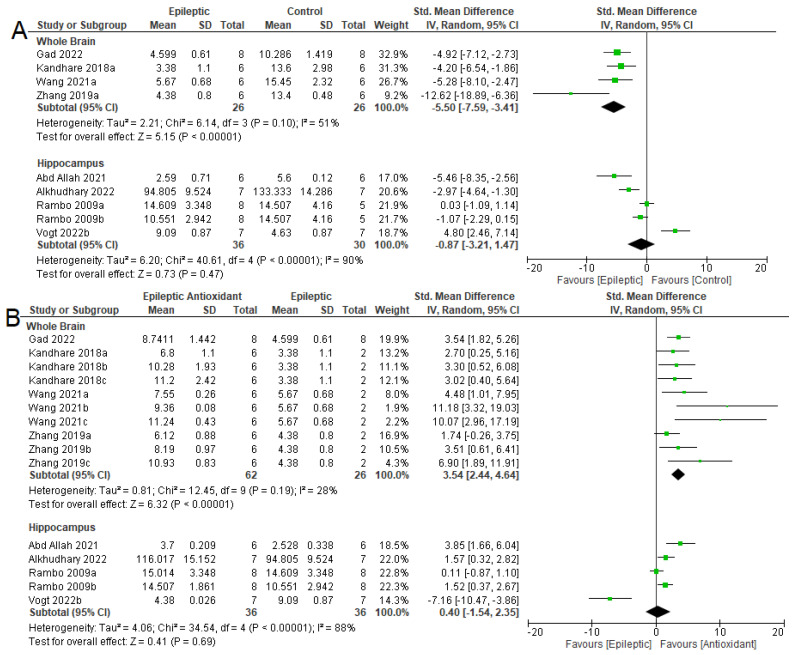
Forest plot comparing SOD activity in different parts of the brain. (**A**) Effect of epilepsy versus control; (**B**) effect of intervention with exogenous antioxidants (epileptics/antioxidants versus epileptics); 95% confidence interval (CI); inverse variance (IV); standard deviation (SD); green square represents effect size. A negative standardized mean difference (SMD) represents lower SOD activity, whereas a positive SMD represents higher SOD activity [54,64,65,67,68,69,70,71].

The SOD activity was reduced in the whole brains of epileptic animals (Figure 5A) (SMD= −5.50 [95% CI: −7.59 to −3.41] *p* < 0.001, I^2^ = 51%), while the use of exogenous antioxidants increased their activity (SMD = 3.54 [95% CI: 2.44 to 4.64] *p* < 0.001, I^2^ = 28%). In the hippocampus, no difference in SOD activity was observed in the two comparisons: epileptic and control (SMD = −0.87 [95% CI: −3.21 to 1.47], *p* = 0.47, I^2^ = 90%) and epileptic without and with the use of antioxidants (SMD = 0.40 [95% CI: −1.54 to 2.35], *p* = 0.69, I^2^ = 88%).

**Figure 6:**
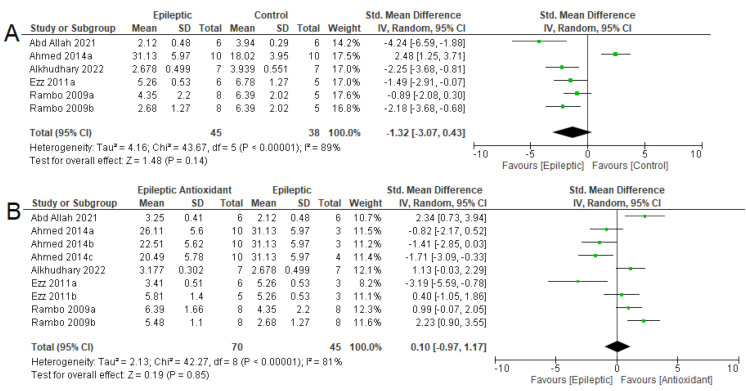
Forest plot comparing CAT activity in different parts of the brain. (**A**) Effect of epilepsy versus control; (**B**) effect of Intervention with exogenous antioxidants (epileptics/antioxidants versus epileptics); 95% confidence interval (CI); inverse variance (IV); standard deviation (SD); green square represents effect size. A negative standardized mean difference (SMD) represents lower CAT activity, whereas a positive SMD represents higher CAT activity [54,57,62,67,70].

CAT was evaluated only in the hippocampus (Figure 6), and there were no differences between epileptic and control animals in the five analyses (SMD = −1.32 [95% CI: −3.07 to 0.43], *p* = 0.14, I^2^ = 89%). There were also no differences between epileptic animals using exogenous antioxidants in the nine analyses (SMD = 0.10 [95% CI: −0.97 to 1.17] *p* = 0.85, I^2^ = 81%).

#### 3.4.3. NKA Activity

NKA activity was assessed in four different regions of the central nervous system of epileptic animals in addition to the whole brain. There was a reduction in 100 % of NKA activity analyses in the whole brain and striatum. In the hippocampus and cortex, there were reductions of 90.9% and 75% in the analyses, respectively. In intervention studies using antioxidants, NKA activity was restored in most of the analyses. In the cortex, striatum, hippocampus, and whole brain, there were increases in NKA activity in 66.7%, 60%, 75%, and 86.4% of the analyses, respectively. In the cerebellar region, only one study on NKA activity was performed, with no difference in either disease exposure or intervention analysis.

**Figure 7:**
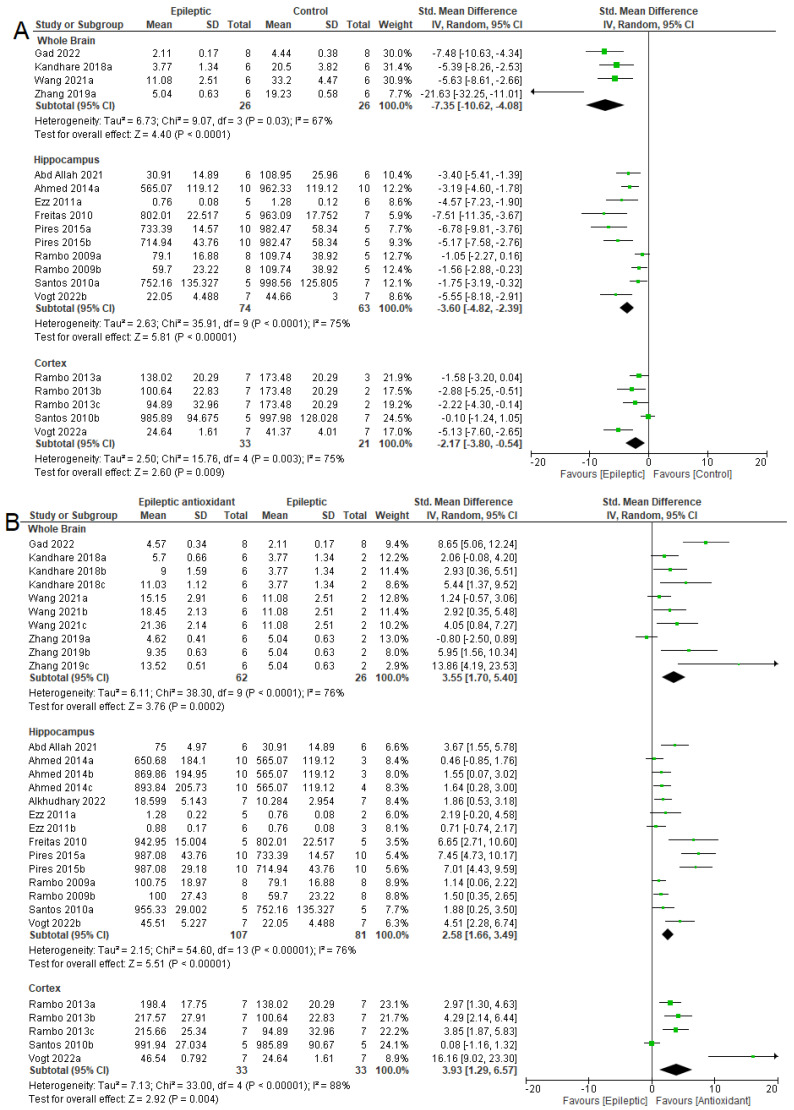
Forest plot comparing NKA activity in different parts of the brain. (**A**) Effect of epilepsy versus control; (**B**) effect of intervention with exogenous antioxidants (epileptics/antioxidants versus epileptics); 95% confidence interval (CI); inverse variance (IV); standard deviation (SD); green square represents effect size. A negative standardized mean difference (SMD) represents lower NKA activity, whereas a positive SMD represents higher NKA activity [20,54,56,57,61,62,63,64,65,67,68,69,70,71].

Meta-analysis (Figure 7A) indicated the effect of epilepsy on NKA activity in these three areas. There was a reduction in NKA activity in the whole brains (SMD = −7.35 [95% CI: −10.62 to −4.08] *p* < 0.0001, I^2^ = 67%), hippocampi (SMD = −3.60 [95% CI: −4.82 to −2.39] *p* < 0.0001, I^2^ = 75%), and cortexes (SMD = −2.17 [95% CI: −3.80 to −0.54] *p* = 0.009, I^2^ = 75%) of epileptic animals when compared to control animals. Figure 7B shows the effects of antioxidant therapy in epileptic animals. There was an increase in NKA activity in all three areas analyzed: the whole brain (SMD = 3.55 [95% CI: 1.70 to 5.40] *p* = 0.0002, I^2^ = 76%), in the hippocampus (SMD = 2.58 [95% CI: 1.66 to 3.49] *p* < 0.00001, I^2^ = 76%), and in the cortex (SMD = 3.93 [95% CI: 1.29 to 6.57] *p* = 0.004, I^2^ = 88%).

### 3.5. Seizure-Related Outcomes and Mortality

**Figure 8:**
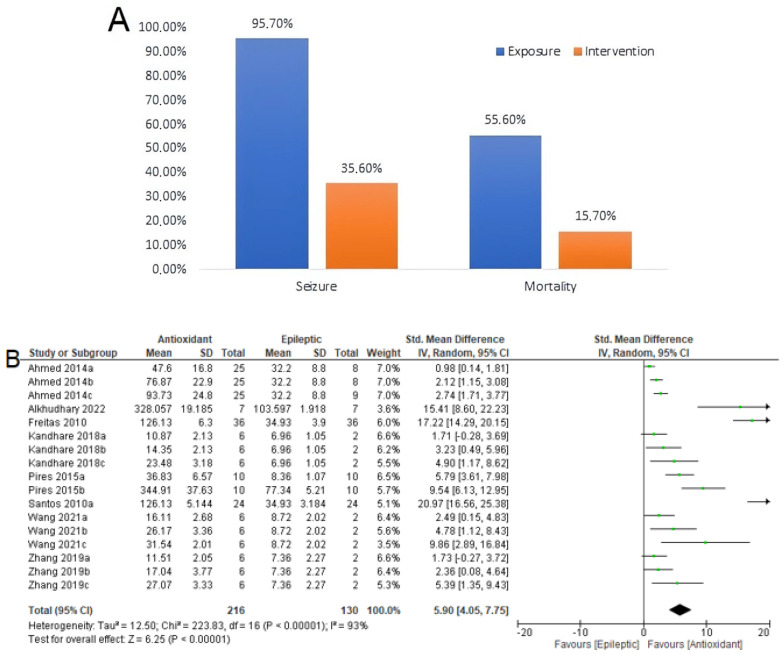
Effect of antioxidant use on seizure-related outcomes and mortality. (**A**) Seizure frequency and mortality for use of antioxidants at all dosages in the primary studies. The data are expressed as percentages. (**B**) Seizure latency. Forest plot comparing antioxidant-treated epileptic animals and untreated epileptic animals. Confidence interval of 95% (CI); inverse variance (IV); standard deviation (SD); green square represents effect size. A negative standardized mean difference (SMD) represents a lower latency, whereas a positive SMD represents a higher latency [20,56,62,63,64,65,68,70].

Seizure-related outcomes included the seizure frequency and latency. The use of antioxidants decreases seizure frequency and mortality in animals with epilepsy. Of the 117 animals that were not treated with the antioxidants (Figure 8A), 112 animals experienced seizures (95.7%). Of the 191 animals that received antioxidant treatment, 68 experienced seizures (35.6%). Among 117 epileptic animals evaluated, 55.6% died of epilepsy. This percentage decreased to 15.7% when epileptic animals received exogenous antioxidants.

Regarding latency (Figure 8B), a meta-analysis was performed on 216 epileptic animals and 130 animals that received exogenous antioxidants. The results (SMD = 5.90 [95% CI: 4.05 to 7.75] *p* < 0.00001, I^2^ = 93%) indicated that the use of exogenous antioxidants increased the seizure latency of epileptic animals. Except for the studies by [62,63], all other studies evaluated the latency period up to 60 min after the induction of epilepsy with PTZ or PILO. Notably, induction occurred between 30 min and 60 min after the use of antioxidants.

### 3.6. Summary of Meta-Analyses

Table 3 summarizes the results of the meta-analysis, indicating whether there was an increase or decrease in the levels or activities of the outcomes. Non-significant data are indicated as n.s.

## 4. Discussion

Epilepsy is a devastating neurological disorder. In this meta-analysis, we demonstrated the neuroprotective effects of exogenous antioxidants in an experimental epilepsy model.

Oxidant agents arise from excitotoxicity resulting from hyperstimulation of the glutamatergic system, observed in epilepsy. Due to the exacerbated increase of glutamate in the synaptic cleft, NMDA (N-methyl-D-aspartate) receptors are excessively stimulated, which causes an accentuated influx of calcium ions. One consequence of this influx of calcium ions into the intracellular environment is the production of NO by neuronal nitric oxide synthase (nNOS), which is linked to the NMDA receptor by the PSD95 protein [72,73]. They can react with superoxides to form peroxynitrite, which causes cell damage [13].

In the present study, there was an increase in NO levels in the brains and hippocampi of epileptic animals compared to control animals, with a possible protective action resulting from the reduction in NO after the use of exogenous antioxidants. Another relevant point of exacerbated NMDA receptor activation is the production of RS, which can occur through NADPH oxidase [73] and metabolic stress in mitochondria [74,75]. NO can react with superoxides from NADPH oxidase and mitochondria to produce peroxynitrite, which can be decomposed to form hydroxyl radicals that are highly toxic to cells [76]. The formation of different RS plays an essential role in lipid peroxidation, which causes rupture of cell membranes, altering permeability, and leading to cell death. MDA is a product of lipid peroxidation [59,64] and has been evaluated using meta-analysis. It was observed that the experimental rodent models of seizure epilepsy used, PILO, KA, and PTZ, showed increased levels of MDA in the brain, hippocampus, and cortex, and antioxidant therapy was able to reverse the increase in MDA levels in epileptic animals in all evaluated areas.

We also found that PCA and RS levels increased in epileptic animals, except in the study by Bortolatto et al. (2011) [58] in cortical areas. According to the authors, this is due to the higher specificity of KA for the hippocampal region, which also influences the data with the use of exogenous antioxidants. Additionally, Wilhelm et al. (2010) [55] used six different dosages of the antioxidant BPD and found no difference in RS levels at the lowest dosage of 1 mg/kg.

In addition to the oxidant factors, it was possible to evaluate the endogenous antioxidant system by meta-analysis by evaluating the levels of GSH and the enzymatic activities of SOD and CAT. There are controversies regarding GSH studies. Some studies have pointed out that the use of antioxidants at low concentrations does not affect GSH levels [62,64,66], whereas Ezz et al. (2011) [57] suggested that they did not increase GSH levels after using the antioxidant NSO due to one of its components, thymoquinone, being reported as being able to cause GSH depletion. Human studies have shown a reduction in total GSH levels in the blood of children with focal epilepsy compared with children without a diagnosis of epilepsy [77]. However, no difference was observed in another prospective study involving 100 patients [78].

This meta-analysis showed that GSH levels were reduced in the brain and hippocampus of epileptic animals and reversed after the use of exogenous antioxidants. These results agreed with those reported by Hussein et al. (2014) [79], who found a reduction in GSH levels in the brains of KA-induced epileptic rats, which was reversed after the use of curcumin. There was also a reduction in GSH levels in the brains of PTZ-induced rats; however, no change in GSH levels was observed after the use of exogenous antioxidants [80]. Regarding GSH levels in the hippocampus, our results were also confirmed by other studies that did not meet the eligibility criteria of this meta-analysis, showing that when the hippocampus is evaluated separately from the rest of the brain there is an increase in GSH after the use of antioxidant therapy [32,81,82,83].

It is known that neuronal GSH synthesis is dependent on the supply of glutamate and cysteine through the EAAC1 transporter found in the neuronal membrane [84]. Furthermore, neuronal GSH synthesis is supported by the supply of cysteine from the cleavage of GSH by astrocytes, which, in turn, depends on the transporters GLAST and GLT-1 to capture glutamate from the synaptic cleft [85]. Under normal conditions, when there is an excessive increase in glutamate in the synaptic cleft, transporters control glutamate levels and transport it to astrocytes and neurons [84,86]. A reduction in the expression of the glutamate transporters GLT-1 and GLAST has already been observed in epileptic animals induced by KA [87] and in a genetic model of epilepsy [88], in addition to verifying lethal spontaneous seizures in GLT-1-deficient mice [89,90]. In this context, failures in GLAST, GLT-1, and EAAC1 transporters in epilepsy, in addition to collaborating with the excitotoxicity of the glutamatergic system, provoke failures in the production of neuronal GSH, justifying the reduction of GSH in epileptic animals observed in the meta-analysis [28,91]. In addition, the results of meta-analyses on GSH indicate that the use of exogenous antioxidants collaborates with the increase in GSH levels which in turn may regulate the glutamatergic system, protecting against excitotoxicity induced by excess glutamate and inhibiting the intracellular influx of calcium ions [92,93].

Superoxide dismutase is another important enzyme in the endogenous antioxidant system that is capable of converting superoxide anions into hydrogen peroxide [94]. This study showed a reduction in SOD activity in the whole brain of epileptic animals compared to that in control animals, which was restored after the use of exogenous antioxidants. These data are in agreement with those of other studies that were not included in the meta-analysis because of eligibility criteria [79,95,96]. In addition to the whole brain, SOD and CAT activities were evaluated in the hippocampus. The meta-analysis showed that there was no difference in the activity of the two enzymes between epileptic animals and controls, or with the use of exogenous antioxidants. The findings of the SOD meta-analysis agree with those of Gao et al. (2014) [97]; however, this contradicts some published studies that showed a reduction in SOD activity in the hippocampi of epileptic animals compared with control animals, while SOD activity was restored after the use of exogenous antioxidants [32,82,98,99,100]. CAT activity is increased [97,98], while other studies point to a reduction in epileptic animals, with CAT activity being reversed after the use of exogenous antioxidants [32,99,100,101].

Both SOD and CAT activities in the hippocampus are expected to be reduced in epileptic animals, since overstimulation of NMDA receptors, in addition to causing an increase in RS production, also causes a reduction in the antioxidant system [93]. Given that GSH plays a key role in controlling RS, it is suggested that there is no synergy in the endogenous antioxidant system in the hippocampus; that is, GSH alone controls RS and does not require the participation of SOD and CAT. There is also the possibility that it is a reward system of the antioxidant system itself; that is, with an increase in oxidant agents, the activity of enzymes is increased. Another point regarding CAT activity is its association with circadian patterns. CAT activity in healthy animals varies over 24 h, with two peaks of activity [102]. This may have influenced the results of the meta-analysis since the primary articles did not show the times when the experimental activities took place. Furthermore, CAT activity is much lower in the brain than in the liver and kidneys [102], which may require more refined technologies to quantify CAT activity.

In the context of increased oxidant factors and the imbalance of antioxidant defenses observed in epilepsy, the study of NKA function is important. NKA plays a fundamental role in neuronal membranes, allowing for the maintenance of the ionic gradient [19]. Preclinical studies have reported that NKA activity is altered in epilepsy models [103], although it remains unchanged in the cerebellum of pilocarpine-induced rats, as suggested by Santos et al. 2010 [56]. In this meta-analysis, it was found that in the whole brain, hippocampus, and cortex, there was a reduction in NKA activity in epileptic animals compared with control animals [104,105,106]. This is because of at least one of the three situations described below. Lipid peroxidation of the plasma membrane is caused by increased MDA levels in epileptic animals. NKA is a transmembrane enzyme. Lipid peroxidation may disrupt the interaction between lipids and NKA, triggering a reduction in enzymatic activity [62], which may occur through nitration of the alpha subunit [105]. Meta-analysis data point to an increase in NO levels in epileptic animals, favoring the chemical reaction between NO and NKA and reducing their activity and sensitivity to reactive species, as NKA has sulfhydryl groups that are potentially oxidized, making the enzyme completely inactivated [107]. To avoid the total inactivity of NKA, there is a reversible process called S-glutathionylation, which consists of the formation of a disulfide bridge between GSH and a cysteine residue of the enzyme. The α-subunit of NKA contains 21 cysteine residues, 12 of which are available for glutathionylation. When glutathionylation occurs in Cys244, enzymatic activity is inhibited whereas in Cys454, Cys456, Cys458, and Cys459, it alters the signaling pathway modulated by NKA [108,109]. Furthermore, the level of glutathionylation has been shown to be dose- and time-dependent, causing complete inhibition of NKA [110], which is also dependent on enzymatic conformation, resulting in maximum glutathionylation in the E1 conformation of the alpha subunit of NKA [109]. Thus, the depletion of GSH observed in epileptic animals in this study may contribute to the reduction in S-glutathionylation, thereby reducing NKA activity.

In addition to the three situations analyzed, another possibility for the reduction in NKA activity in epileptic animals is the substrate ATP. The exacerbated consumption of ATP during convulsions, or even the reduction of its affinity for the enzyme, can lead to its inactivity [105]. This can occur only if S-glutathionylation fails, as this mechanism acts as a switch, preventing NKA from acting at very low ATP levels [110].

Another phenomenon that can occur is that glutamatergic neurotransmission may be impaired as a consequence of NKA malfunction. Previous studies have demonstrated that glutamate transporters and NKA are part of the same macromolecular complex and act synergistically in the regulation of glutamatergic neurotransmission [111,112]. Moreover, glutamate transport activity is regulated by NKA, which depends on sodium ions [113,114]. Selective inhibition of the α2 isoform of NKA is related to lower glutamate uptake [114]. Thus, malfunctioning NKA activity contributes to glutamatergic excitotoxicity as glutamate uptake is compromised [113].

When epileptic animals were treated with exogenous antioxidants, the meta-analysis revealed that changes in NKA activity were reversed in the three areas studied. These data are fundamental, as the normalization of NKA activity in epileptic animals treated with antioxidants provides adequate transport of glutamate to astrocytes, eliminating hyperstimulation of the glutamatergic system. It also induces neuronal GSH production, which is dependent on glutamate uptake by astrocytes and neurons [112]. Thus, the NKA/glutamate transporter complex has the potential to be a therapeutic target for patients with epilepsy.

To assess whether the improvement in biochemical parameters observed through the use of exogenous antioxidants was reflected in functional improvements in epileptic animals, we evaluated parameters such as seizure frequency, seizure latency, and mortality in treated animals. The meta-analysis gathered eight primary studies with 17 analyses and pointed out that the use of exogenous antioxidants increases the latency of seizures in epileptic animals. In addition to the primary studies used in this meta-analysis, several articles have reported similar results [33,54,55,58,60,80,100,115]. To complement the latency seizure data, the current study points to a reduction in both the number of seizures and mortality rate after treatment with external antioxidants. Thus, it is an effective therapy for reducing cell damage and protecting against changes resulting from epilepsy.

There has been an increase in research related to the use of antioxidants for various diseases, including epilepsy, with the objective of improving quality of life. In 2012, Goldberg-Stern et al. [35], in a pilot study, concluded that there was a reduction in the number of daytime seizures in patients receiving melatonin. This was also observed in patients receiving vitamin E [36]; however, patients receiving black seed oil showed no difference compared with those receiving placebo [116].

In summary, OS plays an important role in the development of epileptic seizures by affecting NKA activity and triggering behavioral changes. In view of this, antioxidant therapy undoes OS, normalizing biochemical data, as antioxidants scavenge free radicals, as they can reduce and donate hydrogen ions and mitigate the side effects of free radicals released during lipid peroxidation and cellular damage [71,117]. In addition, exogenous antioxidants are involved in reducing intracellular calcium levels, restoring mitochondrial function, restoring ATP production [62], and inhibiting NADPH oxidase [67].

Thus, antioxidant therapy has an anticonvulsant effect because normalizing the activity of NKA will allow the proper functioning of glutamate transporters, ceasing glutamatergic hyperstimulation, eliminating neuronal toxicity, suppressing seizures [69], and increasing latency [62]. Antioxidants act on the GABAergic system as antiepileptic drugs as they increase the levels of GABA neurotransmitters, ceasing the excitatory process [63,64,68,71,118].

Therefore, we can conclude that antioxidant therapy plays a neuroprotective role in experimental models. In addition, it can act as an adjunct in epilepsy treatment as it interferes with biochemical mechanisms, resulting in an increase in latency and a decrease in seizures and death.

### Study Limitations

Despite an exhaustive search for primary studies, only a small number of studies have evaluated the use of antioxidants in epileptic animals and their association with NKA activity and OS.

The results of this study should be considered within the context of its limitations. One point to be raised is the high risk of bias in primary studies, which highlights a very high detection bias. We do not know if there is any interference with the sex of the animals since all primary studies exclusively used male animals. The high heterogeneity of most meta-analyses performed is due to factors such as different animal models (mice and rats), different types of antioxidants, as well as the duration, dosage, and limited number of studies. In addition, they use different epilepsy-inducing drugs, with variations in the doses of chemoconvulsants. Although we understand that the PILO, KA, and PTZ models are different, we analyze the data together because they show the same trend.

Despite the above, the meta-analyses presented in this article confirm the role of OS in the pathophysiology of epilepsy and its relationship with damage to NKA activity. Furthermore, the use of exogenous antioxidants significantly contributes to the balance between oxidative and antioxidant factors, normalizing NKA activity.

## 5. Conclusions

The results of this meta-analysis showed neural dysfunction in all models of epilepsy and in all brain regions analyzed. There was a decrease in antioxidant defenses such as GSH and SOD, and a decrease in NKA activity. In addition, there was an increase in oxidizing factors, such as NO, as well as an increase in lipid peroxidation markers, which may lead to cell damage. These parameters were associated with seizures and death. In this context, antioxidant therapy reversed the biochemical parameters altered by epilepsy and decreased the occurrence of seizures and animal mortality. Our findings confirm the neuroprotective effects of antioxidant therapy in epilepsy.

## Figures and Tables

**Table 1:** Risk of bias assessment using SYRCLE’s risk of bias tool for animal studies.

Study	Q1	Q2	Q3	Q4	Q5	Q6	Q7	Q8	Q9	Q10
Oliveira 2004 [51]	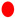	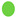	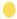	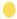	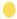	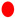	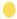	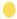	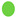	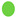
Fighera 2006 [52]	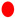	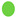	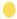	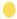	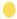	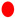	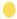	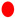	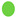	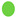
Wilhelm 2009 [53]	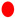	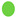	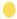	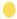	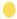	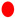	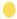	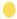	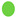	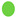
Rambo 2009 [54]	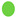	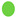	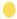	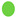	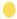	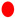	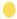	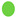	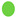	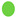
Wilhelm 2010 [55]	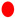	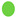	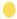	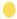	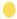	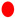	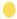	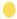	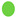	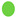
Freitas 2010 [20]	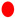	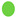	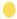	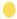	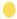	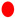	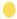	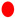	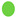	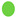
Santos 2010 [56]	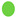	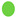	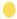	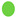	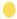	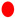	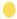	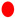	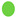	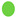
Ezz 2011 [57]	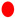	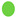	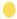	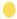	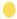	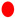	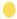	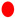	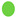	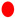
Bortolatto 2011 [58]	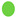	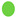	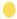	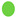	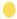	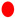	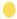	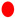	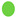	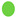
Souza 2013 [59]	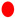	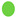	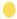	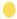	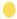	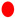	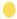	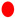	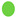	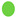
Della-Pace 2013 [60]	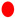	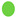	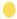	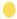	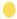	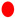	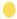	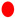	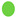	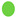
Rambo 2013 [61]	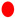	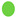	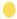	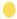	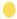	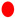	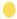	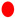	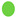	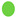
Ahmed 2014 [62]	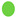	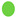	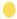	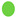	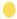	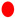	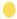	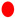	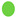	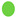
Pires 2015 [63]	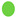	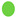	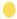	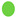	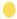	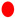	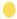	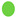	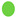	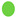
Kandhare 2018 [64]	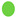	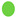	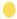	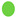	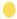	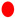	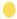	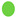	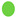	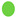
Zhang 2018 [65]	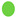	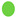	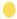	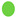	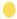	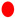	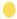	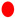	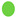	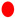
Tao 2020 [66]	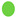	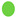	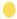	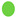	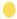	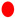	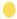	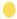	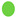	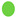
Abd Allah 2021 [67]	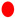	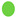	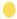	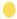	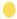	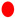	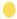	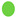	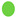	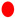
Wang 2021 [68]	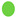	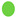	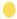	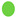	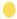	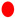	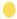	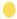	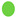	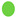
Vogt 2022 [69]	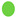	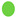	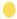	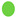	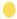	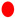	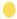	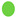	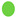	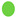
Alkhudhary 2022 [70]	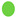	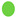	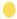	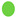	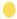	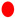	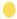	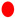	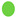	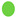
Gad 2022 [71]	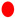	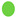	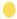	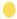	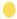	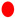	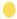	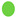	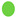	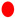

Note: low risk of bias (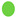); high risk of bias (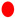); unclear risk of bias (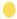). Questions Q1, Q2, and Q3 relate to selection bias; Q4 and Q5 relate to performance bias; Q6 and Q7 relate to detection bias; and questions Q8, Q9, and Q10 relate to attrition, information, and other biases, respectively. Details are provided in Supplementary Data N° 1.

**Table 2:** Description of the animals, antioxidants and biochemical outcomes of the epilepsy and epilepsy + antioxidants studies.

Reference	Brain Areas Sample Size	Animal Model Characteristics	Antioxidant	Results
* Oliveira et al., 2004 [51]	Striatum 10–14	Wistar Rats/270–300 g PTZ 1.8 μmol/2 μL Intraestrial	Ascorbic acid (30, 100 or 300 mg/Kg)/i.p. 30 min before PTZ	Epilepsy: NKA ↓; PCA ↑ Epilepsy + antioxidants: NKA ↑ (1); n.s (2); PCA ↓ (1); n.s (2)
* Fighera et al., 2006 [52]	Striatum 6–8	Wistar Rats/270–300 g PTZ 1.8 µmol/2 µL Intraestrial	GM1 ganglioside 50 mg/kg/i.p. 30 min before the injection of PTZ	Epilepsy: NKA ↓;PCA ↑; MDA ↑ Epilepsy + antioxidants: NKA ↑; PCA ↓; MDA ↓
* Wilhelm et al., 2009 [53]	Whole Brain 8–12	Wistar Rats/40–50 g PILO 400 mg/Kg/i.p.	3-ASP (10, 25 or 50 mg/Kg)/Oral 30 min before of PILO	Epilepsy: NKA ↓; GPx ↓; GST ↑; SOD ↓; CAT ↑; RS ↑ Epilepsy + antioxidants: NKA ↑; GPx n.s; GST n.s; SOD ↑ (1)/n.s (2); CAT ↓ (2)/n.s (1); RS ↓
Rambo et al., 2009 [54]	Hippocampus 8–10	Wistar Rats/250–300 g PTZ (30 or 60 mg/Kg)/i.p.	Creatine 300 mg/Kg Oral gavage 6 weeks	Epilepsy: NKA ↓; SOD n.s; CAT ↓ (1)/n.s (1); MDA ↑; PCA ↑ Epilepsy + antioxidants: NKA ↑; SOD ↑ (1)/n.s (1); CAT ↑ (1)/n.s (1); MDA ↓; PCA ↓ (1)/n.s
* Wilhelm et al., 2010 [55]	Whole Brain 5–8	Swiss Mice/25–35 g PILO 400 mg/Kg/i.p.	BPD (1, 5, 10, 25, 50 or 100 mg/Kg) Oral gavage 30 min before PILO	Epilepsy: NKA ↓; GPx ↓; GST ↑; CAT ↑; MDA↑; RS ↑ Epilepsy + antioxidants: NKA ↑; GPx n.s; GST ↑(1)/n.s (5); CAT n.s; MDA ↓ (4)/n.s (2); RS ↓ (5)/n.s (1)
Freitas, 2010 [20]	Hippocampus 5–7	Wistar Rats/250–280 g PILO 400 mg/Kg/i.p.	LA 10 mg/Kg/i.p./30 min before PILO	Epilepsy: NKA ↓; GPx ↑; GSH ↓; GR n.s Epilepsy + antioxidants: NKA ↑; GPx ↑; GSH ↑; GR n.s
Santos et al., 2010 [56]	Hippocampus 5–7	Wistar Rats/250–280 g PILO 400 mg/Kg/i.p.	LA 10 mg/Kg/i.p./30 min before PILO	Epilepsy: NKA ↓ Epilepsy + antioxidants: NKA ↑
	Striatum 5–7			Epilepsy: NKA ↓ Epilepsy + antioxidants: NKA ↑
	Cortex 5–7			Epilepsy: NKA n.s Epilepsy + antioxidants: NKA n.s
	Cerebellum 5–7			Epilepsy: NKA n.s Epilepsy + antioxidants: NKA n.s
Ezz et al., 2011 [57]	Hippocampus 5–7	Wistar Rats/200–250 g PILO 380 mg/Kg/i.p.	Curcumin 80 mg/Kg or Nigella sativa oil (NSO) 4 mL/Kg/Orally/21 days	Epilepsy: NKA ↓; GSH ↓; CAT ↓; NO ↑; MDA n.s Epilepsy + antioxidants: NKA and GSH: ↑ (1)/n.s (1); CAT and NO: ↓ (1)/n.s (1); MDA n.s
* Bortolatto et al., 2011 [58]	Hippocampus 8–10	Wistar Rats/200–300 g KA 10 mg/Kg/i.p.	DTDS (50 or 100 mg/Kg)/Oral by gavage 1 h after the animals received KA	Epilepsy: NKA ↑; GPx n.s; PCA ↑; RS ↑ Epilepsy + antioxidants: NKA ↓; GPx n.s; PCA ↓; RS ↓
	Cortex 8–10			Epilepsy: NKA ↑; GPx, PCA and RS: n.s Epilepsy + antioxidants: NKA ↓; GPx, PCA and RS: n.s
* Souza et al., 2013 [59]	Cortex 7–8	Wistar Rats/270–300 g PTZ 60 mg/Kg/i.p.	Caffeine 6 mg/Kg/Oral by gavage 60 min before PTZ	Epilepsy: NKA ↓; GSH ↓; MDA↑ Epilepsy + antioxidants: NKA ↑; GSH ↑; MDA ↓
* Della-Pace et al., 2013 [60]	Cortex 8–9	Swiss Mice/25–35 g PTZ 80 mg/Kg/i.p.	TTHL 30 mg/Kg/Orally by gavage/60 min before PTZ	Epilepsy: NKA ↓; MDA↑; PCA ↑ Epilepsy + antioxidants: NKA ↑; MDA ↓; PCA ↓
Rambo et al., 2013 [61]	Cortex 5–7	Wistar Rats/270–300 g PTZ (30, 45 or 60 mg/Kg)/i.p.	Creatine 300 mg/Kg/Orally/ 45 min before PTZ	Epilepsy: NKA ↓; MDA↑; PCA ↑ Epilepsy + antioxidants: NKA ↑; MDA ↓; PCA ↓
Ahmed, 2014 [62]	Hippocampus 10	Sprague-Dawley Rats/250–280 g PILO 400 mg/Kg/i.p.	Idebenone (50, 100 or 200 mg/kg) i.p./ 3 successive days	Epilepsy: NKA ↓; GSH ↓; CAT ↑; NO ↑; MDA↑ Epilepsy + antioxidants: NKA ↑ (2)/n.s (1); GSH ↑ (1)/n.s (2); CAT, NO and MDA: ↓ (2)/n.s (1)
Pires et al., 2015 [63]	Hippocampus 10	Swiss Mice/25–30 g/PTZ 60 mg/Kg or PILO400 mg/Kg/i.p	CA 100 mg/Kg/i.p./30 min before PILO or PTZ	Epilepsy: NKA ↓ Epilepsy + antioxidants: NKA ↑
Kandhare et al., 2018 [64]	Whole Brain 6	Swiss Mice/18–22 g/PTZ 90 mg/Kg/i.p.	Morin (10, 20 or 40 mg/Kg)/i.p./45 min before PTZ	Epilepsy: NKA ↓; GSH ↓; SOD ↓; NO ↑; MDA↑ Epilepsy + antioxidants: NKA and GSH: ↑ (2)/n.s (1); SOD ↑; NO and MDA: ↓ (2)/n.s (1)
Zhang et al., 2018 [65]	Whole Brain 6	Swiss Mice/18–22 g/PTZ 90 mg/Kg/i.p.	TEMPOL (50, 100 or 200 mg/Kg)/Oral 45 min before PTZ	Epilepsy: NKA ↓; GSH ↓; SOD ↓; NO ↑; MDA ↑ Epilepsy + antioxidants: NKA ↑ (2)/n.s (1); GSH ↑; SOD ↑; NO and MDA: ↓ (2)/n.s (1)
* Tao et al., 2020 [66]	Whole Brain 5–6	Swiss Mice/18–22 g PTZ 90 mg/Kg/i.p.	PA (50, 100 or 200 mg/Kg)/i.p. 45 min before PTZ	Epilepsy: NKA ↓; GSH ↓; SOD ↓; NO ↑; MDA↑ Epilepsy + antioxidants: NO and MDA: ↓ (2)/n.s (1); NKA, GSH and SOD: ↑ (2)/n.s (1)
Adb Allah et al., 2021 [67]	Hippocampus 6	Wistar Rats/150–170 g PILO 300 mg/kg/i.p.	M. officinalis extract (MOE)/250 mg/Kg Oral for 2 weeks	Epilepsy: NKA ↓; SOD ↓; CAT ↓; MDA↑ Epilepsy + antioxidants: NKA ↑; SOD ↑; CAT ↑; MDA ↓
Wang et al., 2021 [68]	Whole Brain 6	Swiss Mice/20–30 g PTZ 70 mg/Kg/i.p.	EE-ATF (50, 75 or 100 mg/Kg)/Oral 30 min before PTZ	Epilepsy: NKA ↓; GSH ↓; SOD ↓; NO ↑; MDA ↑ Epilepsy + antioxidants: NKA ↑; SOD ↑; NO ↓; MDA ↓
Vogt et al., 2022 [69]	Hippocampus 7	Swiss Mice/25–35 g PTZ 35 mg/Kg/i.p.	QTCA-1 10 mg/kg by gavage 30 min before PTZ	Epilepsy: NKA ↓; SOD ↑; MDA ↑; RS ↑ Epilepsy + antioxidants: NKA ↑; SOD ↓; MDA ↓; RS ↓
	Cortex 7			Epilepsy: NKA ↓; SOD ↑; MDA↑; RS ↑ Epilepsy + antioxidants: NKA ↑; SOD ↓; MDA ↓; RS ↓
Alkhudhary et al., 2022 [70]	Hippocampus 7	Wistar Rats/180–200 g PTZ 60 mg/Kg/i.p.	ESE (250 mg/Kg)/Oral/for 7 days	Epilepsy: NKA ↓; GSH ↓; SOD ↓; CAT ↓; GPx ↓; GR↓; NO ↑; MDA ↑ Epilepsy + antioxidants: NKA ↑; GSH ↑; SOD ↑; CAT ↑; GPx ↑; GR ↑; NO ↓; MDA ↓
Gad et al., 2022 [71]	Whole Brain 8	Sprague-Dawley Rats/150–180 g/PILO 300 mg/Kg/i.p.	Passiflora extract 200 mg/Kg/intragastric intubation/4 weeks	Epilepsy: NKA ↓; SOD ↓; CAT ↓; GR ↓; MDA↑ Epilepsy + antioxidants: NKA ↑; SOD ↑; CAT ↑; GR ↑; MDA n.s

Note: Carvacryl acetate (CA); 1-(7-chloroquinolin-4-yl)-5-methyl-N-phenyl-1H-1,2,3-triazole-4-carboxamide (QTCA-1); Ethanolic extract of *A.tsaoko* fruits (EE-ATF); 3-alkynyl selenophene (3-ASP); (E)-2-benzylidene-4-phenyl-1,3-diselenole (BPD); 4-Hydroxy-2,2,6,6-tetramethylpiperidine-N-oxyl (TEMPOL); *Phyllathin amarus* extract (PA); *Echinops spinosus* extract (ESE); Lipoic acid (LA); TTHL (Triterpene 3β, 6β, 16β-trihidroxilup-20(29)-ene); DTDS (2,2′-Dithienyl diselenide); intraperitoneal (i.p.). * Studies excluded from the meta-analysis; ↑ (significant increase); ↓ (significant decrease); n.s. (non-significant data), considering *p* < 0.05.

**Table 3:** Summary of meta-analyses.

Outcomes	Brain Part	Epileptics	Epileptics/Antioxidant
GSH	WB, HIP	↓	↑
SOD	WB, HIP	↓, n.s	↑, n.s
CAT	HIP	n.s	n.s
NO	WB, HIP	↑	↓
MDA	WB, HIP, COR	↑	↓
NKA	WB, HIP, COR	↓	↑
Latency			↑

Note: ↑ (significant increase); ↓ (significant decrease); n.s (non-significant data), considering *p* < 0.05. Whole brain (WB), hippocampus (HIP), cortex (COR), Epileptics (untreated epileptic animals), Epileptics/Antioxidant (epileptic animals using antioxidant therapy).

## Data Availability

Published systematic review and PROSPERO (CRD42022356960).

## Supplementary Materials

The following supporting information can be downloaded at: https://www.mdpi.com/article/10.3390/antiox12071397/s1, Supplementary Data N° 1: Risk of bias assessment; Supplementary Data N° 2: The brain area under study and the outcome assessed.
