# Supplementary material for: Antioxidant Therapy Reduces Oxidative Stress, Restores Na,K-ATPase Function and Induces Neuroprotection in Rodent Models of Seizure and Epilepsy: A Systematic Review and Meta-Analysis
Source: Antioxidants (Basel). 2023 Jul 7;12(7):1397. doi: 10.3390/antiox12071397 (PMC10376594; doi:10.3390/antiox12071397)
Supplement: Supplementary file 1 [file antioxidants-12-01397-s001.zip › antioxidants-2372509-supplementary.pdf]

Supplementary Data N°1: Risk of bias assessment

Selection Bias – Q1: Sequence of Allocation: Does the study indicate that the control, epileptic, and epileptic/antioxidant groups were randomly allocated? Q2: Baseline Characteristics: Are sex, age, and weight of the animals similar between the groups? Q3: Allocation concealment: Was the induction of epilepsy blinded; that is, was there allocation concealment of epileptic animals at the time of induction?

Performance Bias - Q4: Random housing: Does the article describe whether the control, epileptic, and epileptic/antioxidant groups were randomly distributed among housing exposed to the same conditions: same vivarium in standardized boxes, light/dark cycle, temperature, and feeding? Q5: Blinding: Does the study describe whether the investigator was aware of which animals received each type of intervention (saline or antioxidant) during the study?

Detection bias: Q6, Randomized outcome assessment: Does the study describe whether the outcome assessment of the groups was performed randomly? Q7: Blinding of the outcome assessor: Was the investigator aware of which animals had received each type of intervention (saline or antioxidant) in the outcome assessment?

Attrition Bias – Q8: Incomplete outcome: did the study exclude animals in the outcome assessment?

.Information Bias Q9: Selective outcome reporting: Was there selective reporting of outcomes in the study when comparing the methodology and results sections?

Other sources of bias Q10: Was there an evaluation of epileptic seizures after induction in animals?

Supplementary Data N° 2: The brain area under study and the outcome assessed.

Outcomes	Brain part	Articles
reactive species	Whole Brain	*Wilhelm et al., 2009<br>*Wilhelm et al., 2010
reactive species	hippocampus	*Bortolatto et al., 2011<br>Vogt et al., 2022
reactive species	Cortex	*Bortolatto et al., 2011<br>Vogt et al., 2022
protein carbonyl	hippocampus	Rambo et al., 2009<br>*Bortolatto et al., 2011
protein carbonyl	Cortex	*Bortolatto et al., 2011<br>*Della-Pace et al., 2013<br>Rambo et al., 2013
protein carbonyl	Striatum	*Oliveira et al., 2004<br>*Fighera et al., 2006
Nitric oxide	Whole Brain	Kandhare et al., 2018<br>Zhang et al., 2019<br>*Tao et al., 2020<br>Wang et al., 2021
Nitric oxide	hippocampus	Ezz et al., 2011<br>Ahmed, 2014<br>Alkhudhary et al., 2022
malondialdehyde	Whole Brain	*Wilhelm et al., 2010<br>Kandhare et al., 2018<br>Zhang et al., 2019<br>*Tao et al., 2020<br>Wang et al., 2021<br>Gad et al., 2022
malondialdehyde	hippocampus	Rambo et al., 2009<br>Ezz et al., 2011<br>Ahmed, 2014<br>Abd Allah et al., 2021<br>Vogt et al., 2022<br>Alkhudhary et al., 2022
malondialdehyde	Cortex	*Souza et al., 2013<br>*Della-Pace et al., 2013<br>Rambo et al., 2013<br>Vogt et al., 2022
malondialdehyde	Striatum	*Fighera et al., 2006
glutathione S-transferase	Whole Brain	*Wilhelm et al., 2009<br>*Wilhelm et al., 2010
glutathione peroxidase	Whole Brain	*Wilhelm et al., 2009<br>*Wilhelm et al., 2010
glutathione peroxidase	hippocampus	Freitas 2010<br>*Bortolatto et al., 2011<br>Alkhudhary et al., 2022
glutathione peroxidase	Cortex	*Bortolatto et al., 2011
glutathione reductase	Whole Brain	Gad et al., 2022

glutathione reductase	hippocampus	Freitas 2010<br>Alkhudhary et al., 2022
reduced glutathione	Whole Brain	Kandhare et al., 2018<br>Zhang et al., 2019<br>*Tao et al., 2020<br>Wang et al., 2021
reduced glutathione	hippocampus	Freitas 2010<br>Ezz et al., 2011<br>Ahmed, 2014<br>Alkhudhary et al., 2022
reduced glutathione	Cortex	*Souza et al., 2013
superoxide dismutase	Whole Brain	*Wilhelm et al., 2009<br>Kandhare et al., 2018<br>Zhang et al., 2019<br>*Tao et al., 2020<br>Wang et al., 2021<br>Gad et al., 2022
superoxide dismutase	hippocampus	Rambo et al., 2009<br>Abd Allah et al., 2021<br>Vogt et al., 2022b<br>Alkhudhary et al., 2022
superoxide dismutase	Cortex	Vogt et al., 2022a
catalase	Whole Brain	*Wilhelm et al., 2009<br>*Wilhelm et al., 2010<br>Gad et al., 2022
catalase	hippocampus	Rambo et al., 2009<br>Ezz et al., 2011<br>Ahmed, 2014<br>Abd Allah et al., 2021<br>Alkhudhary et al., 2022
Na,K-ATPase	Whole Brain	*Wilhelm et al., 2009<br>*Wilhelm et al., 2010<br>Kandhare et al., 2018<br>Zhang et al., 2019<br>*Tao et al., 2020<br>Wang et al., 2021<br>Gad et al., 2022
Na,K-ATPase	hippocampus	Rambo et al., 2009<br>Freitas 2010<br>Santos et al., 2010<br>Ezz et al., 2011<br>*Bortolatto et al., 2011<br>Ahmed, 2014<br>Pires et al., 2015<br>Abd Allah et al., 2021<br>Vogt et al., 2022<br>Alkhudhary et al., 2022
	Cortex	Santos et al., 2010<br>*Bortolatto et al., 2011<br>*Souza et al., 2013

Na,K-ATPase		*Della-Pace et al., 2013<br>Rambo et al., 2013<br>Vogt et al., 2022
Na,K-ATPase	Striatum	Oliveira et al., 2004<br>*Fighera et al., 2006<br>Santos et al., 2010
Na,K-ATPase	cerebellum	Santos et al., 2010

Note: The data regarding GSH levels from the study by Abd Allah et al. (2021) were excluded due to data inconsistency because the figure had different information compared to the discussion in the article. The author admitted that there was an error in figure 3B and did not make the data available by email [20, 51-71].

* Articles included only in the systematic review.
